# An Experimental and Numerical Investigation on Bubble Growth in Polymeric Foams

**DOI:** 10.3390/e24020183

**Published:** 2022-01-26

**Authors:** Daniele Tammaro, Massimiliano M. Villone, Gaetano D’Avino, Pier Luca Maffettone

**Affiliations:** Dipartimento di Ingegneria Chimica, dei Materiali e della Produzione Industriale, Università degli Studi di Napoli Federico II, Piazzale V. Tecchio, 80, 80125 Napoli, Italy; daniele.tammaro@unina.it (D.T.); gadavino@unina.it (G.D.); p.maffettone@unina.it (P.L.M.)

**Keywords:** gas foaming, bubble growth, experiments, direct numerical simulations, single bubble growth model

## Abstract

The cellular morphology of thermoplastic polymeric foams is a key factor for their performances. Three possible foam morphologies exist, namely, with closed cells, interconnected cellular structure, and open cells. In the gas foaming technology, a physical blowing agent, e.g., ${\mathrm{CO}}_{2}$ or $N_{2}$, is used to form bubbles at high pressure in softened/melted polymers. As a consequence of a pressure quench, the bubbles grow in the liquid matrix until they impinge and possibly break the thin liquid films among them. If film breakage happens, the broken film may retract due to the elastic energy accumulated by the polymeric liquid during the bubble growth. This, in turn, determines the final morphology of the foam. In this work, we experimentally study the growth of ${\mathrm{CO}}_{2}$ bubbles in a poly(e-caprolactone) (PCL) matrix under different pressure conditions. In addition, we perform three-dimensional direct numerical simulations to support the experimental findings and rationalize the effects of the process parameters on the elastic energy accumulated in the liquid at the end of the bubble growth, and thus on the expected morphology of the foam. To do that, we also extend the analytic model available in the literature for the growth of a single bubble in a liquid to the case of a liquid with a multi-mode viscoelastic constitutive equation.

## 1. Introduction

Due to their mechanical, transport, acoustic, and impact absorption properties, polymeric foams are used in a multitude of different applications, e.g., transportation, construction, packaging, food, extraction and separation, tissue engineering, leisure, and sport [1,2]. The physical gas foaming technology, which makes use of a physical blowing agent (such as carbon dioxide or nitrogen) to form bubbles in softened/melted polymers, is the most widely employed process for the making of polymeric foams, mainly because of its high productivity [1,3]. Depending on their morphology, foams can be classified as ‘closed-cell’ (with bubbles separated by walls made of the polymeric material), with ‘interconnected cellular structure’ (having mostly closed cells with controlled interconnections in between them), and ‘open-cell’ (with no walls at all and the polymer solely confined to cell struts), and their distinctive features and performances derive from their internal pore morphology [4,5,6].

The sequence of operations involved in the gas foaming technology is the following [1]: (1) blowing gas solubilization (yielding a polymer–gas solution); (2) bubble nucleation induced by an instantaneous pressure quench or temperature rise; (3) bubble growth; (4) foam setting. These operations are interconnected and their interplay strongly affects the cell morphology and, thus, the properties of the final product. For instance, the number of bubbles nucleating in step 2 grows exponentially with the amount of blowing gas solubilized in step 1 [7]. In addition, for polymers that have the ability to crystallize, the bubble growth in step 3 and foam setting in step 4 depend on the amount of solubilized blowing gas because of the plasticization effect that this has on the polymer and on its influence on polymer crystallization rate and temperature [8]. The interconnection among the technological operations makes it complicated to optimize the foaming process by acting on the two most powerful leverages, namely, temperature and pressure. For example, changing the foaming temperature modifies the solubility and the diffusivity of the blowing agent, the liquid-gas interfacial properties, and the rheological behavior of the mixture. Although, in principle, it is possible to make measurements to quantify such properties, it is very challenging to predict their combined influence on a foam, especially under transient conditions.

In view of the increasingly urgent ecological transition, poly(e-caprolactone) (PCL) represents a promising alternative to polymers of fossil origin due to its biodegradability. In addition, it is particularly suitable for biomedical applications, such as the fabrication of scaffolds. At the same time, the knowledge on the behavior of this material is still somehow limited. For these reasons, in this paper we aim to improve the understanding of the influence that process conditions, in particular gas pressure, have on the density of foams made by using PCL as the matrix liquid and carbon dioxide as the physical blowing agent. To do that, we perform experiments at different foaming pressures on a simplified system, i.e., a homemade apparatus with a visualization window designed for microcellular foaming. In order to compare with experimental data and to further investigate quantities that are hardly attainable experimentally, e.g., the stress fields in the polymeric liquid, we perform three-dimensional direct numerical simulations of bubble growth in a viscoelastic liquid with the finite element method. Recently, the finite element method has been employed to simulate the growth of gas bubbles in Newtonian liquids both in 2D and in 3D [9,10,11], yet, to the best of our knowledge, this is the first time that bubble dynamics in a viscoelastic liquid is studied computationally in 3D. As an input to the simulations, it is required to provide a suitable bubble growth law, which is derived by extending the analytic model for the growth of a single bubble in a liquid available in the literature [6,12] to the case of a liquid with a multi-mode viscoelastic constitutive equation.

## 2. Experiments

### 2.1. Materials

**Figure 1:**
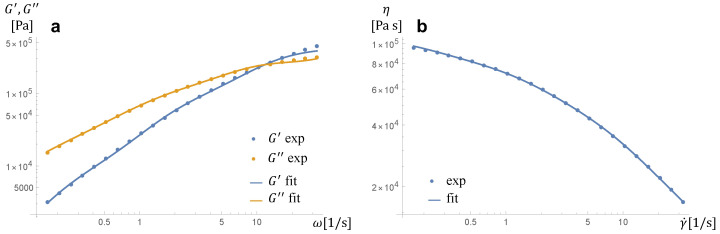
Linear viscoelastic moduli (**a**) and shear viscosity (**b**) of PCL at 35 °C. The symbols represent the experimental measurements, the lines represent the predictions of a three-mode Giesekus constitutive equation whose parameters are reported in Table 1.

Poly(e-caprolactone) PCL Capa 6800 with a melt flow index of 3.02 g/10 min at a temperature of 35 °C and weight load of 2.16 kg, weight average molecular weight of 120 kDa, and number average molecular weight of 69 kDa has been supplied by Perstrop Holding, Sweden. The storage and loss moduli of the polymer and its shear viscosity have been measured under a nitrogen atmosphere using a strain-controlled Rheometric Scientific ARES rheometer (TA Instruments, New Castle, DE, USA) with parallel plates having a diameter of 25 mm and a gap of about 1 mm. The strain amplitude has been set large enough to detect a reliable signal while keeping the measurement in the linear regime, i.e., between 1 and 10%. Time sweep tests have been performed before the frequency sweep in order to measure the stability of the sample. The rheological data of the polymer at 35 °C, i.e., the temperature at which the experiments reported in this work have been done, are given in Figure 1.

${\mathrm{CO}}_{2}$ (99.95% pure) supplied by Sol Group S.p.A., Italy, was used as the physical blowing agent.

### 2.2. Foaming Equipment and Procedure

**Figure 2:**
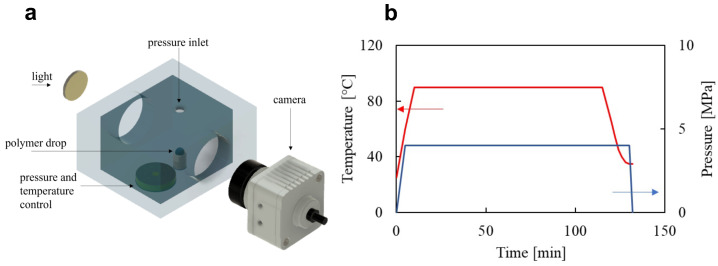
(**a**) 3D rendering of the experimental foaming apparatus. (**b**) Temperature (red) and pressure (blue) temporal histories during the foaming process.

Based on an existing foaming visualization device [13,14], we have designed and realized an experimental apparatus, shown in Figure 2a, composed of a pressurized vessel for the execution of the gas foaming process and of a system to allow its visualization. Experiments are conducted by using the following procedure: a spherical-shape PCL pellet with a mass of around 10 mg is gently laid on a substrate in the middle of the vessel, taking care to ensure that the substrate is level; polymeric pellets are saturated with ${\mathrm{CO}}_{2}$ for 4 h at 80 °C and saturation pressure $p_{\mathrm{sat}}$, as shown in Figure 2b; after saturation, the vessel is cooled to the foaming temperature $T_{\mathrm{foam}}=35$ °C with a controlled, repeatable cooling history; at the foaming temperature, the sample is pressure-quenched to ambient pressure with a pressure drop rate (PDR) in the order of 10 MPa s^−1^ [15,16]. It is worth remarking that, given the volume of the pellet, the sphere is the geometrical shape that minimizes the external surface area, thus the blowing agent lost by skin effect [17]. In addition, the saturation temperature and time are chosen such as to ensure the complete softening of the polymeric pellet and the complete solubilization of the blowing agent. The experiments are performed at the same value of $T_{\mathrm{foam}}$ and at three values of the saturation pressure, i.e., $p_{\mathrm{sat}}=4,5,6\;M\mathrm{Pa}$. The substrate on which the pellet is deposed is a horizontally-oriented cylinder placed in the middle of a closed metallic chamber with four ports based on 1/2” NPT threads. Two ports are sight windows in line with the drop to observe the foaming, then there is one venting port for gas injection through a syringe pump and aspiration through a vacuum pump and one port dedicated to a platinum-resistance thermometer (Pt100 with 3 wires) for measuring the temperature as close as possible to the polymer sample. The cylinder is heated by two electrical tapes placed in contact with its upper and lower surfaces. The temperature control is provided by a fuzzy thermo-regulator (Ascon-New England Temperature Solutions, Attleboro, MA, model X1) and recorded via software on a computer. The evolution of the process is captured by a DMK 41AUO2 camera, Germany, while the background is illuminated by a diffused light (as shown in Figure 2a). The images are recorded and analyzed through MATLAB to evaluate the expansion of the polymeric pellet over time. The images are binarized by using the ‘im2bw’ function and the pellet profile is traced by using the ‘bwboundaries’ function. The volume of the expanding polymeric pellet is calculated by assuming it has an axisymmetric shape.

### 2.3. Experimental Results

**Figure 3:**
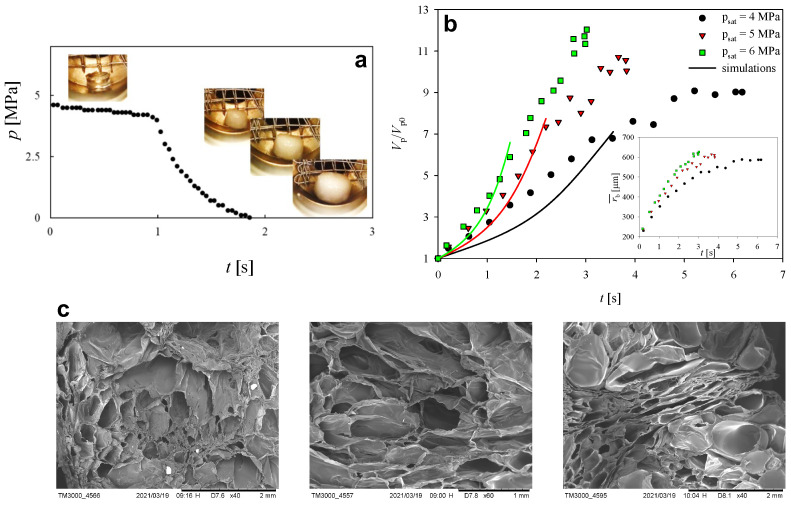
(**a**) Experimental temporal evolution of the pressure inside the vessel at $T_{\mathrm{foam}}=35$ °C and $p_{\mathrm{sat}}=5$ MPa, also showing snapshots of the growing foamed pellet. The origin of the horizontal axis is placed 1 s before the pressure-quench. (**b**) Experimental (symbols) and numerical (lines) temporal histories of the pellet/domain volume *V*, normalized by the initial value $V_{0}$, during the foaming process at $T_{\mathrm{foam}}=35$ °C and $p_{\mathrm{sat}}=4,5,6$ MPa (see legend). Inset: experimental temporal histories of the average bubble radius ${\bar{r}}_{b}$. (**c**) Examples of SEM images of the experimental foam morphology at $T_{\mathrm{foam}}=35$ °C and $p_{\mathrm{sat}}=4,5,6$ MPa (from left to right).

In Figure 3a, we report the experimental temporal evolution of the pressure inside the vessel at $T_{\mathrm{foam}}=35{\;}^{\circ }C$ and $p_{\mathrm{sat}}=5\;M\mathrm{Pa}$. The origin of the horizontal axis is placed 1 s before the pressure-quench. In this regard, it can be remarked that a slight pressure decrease happens during the saturation stage, thus, at the beginning of the pressure-quench, the pressure inside the vessel is slightly lower than the nominal $p_{\mathrm{sat}}$-value. At t=1s, a pressure drop to ambient pressure is imposed, lasting almost 1 s. Figure 3a also shows a snapshot of the saturated PCL pellet before the pressure-quench (in the upper left corner) and three snapshots taken while the pressure is decreasing, showing a progressive increase of the volume of the pellet due to the growth of ${\mathrm{CO}}_{2}$ bubbles triggered by pressure decrease. In Figure 3b, the symbols represent the experimental temporal evolution of the normalized pellet volume $V/V_{0}$ ($V_{0}$ being the pellet volume prior to the beginning of bubble growth) at $T_{\mathrm{foam}}=35{\;}^{\circ }C$ and $p_{\mathrm{sat}}=4,5,6\;M\mathrm{Pa}$, as reported in the legend. It is apparent that, at increasing $p_{\mathrm{sat}}$, *V* increases faster and to a larger final value. This can be connected to the larger amount of ${\mathrm{CO}}_{2}$ that is solubilized into the PCL pellet at larger $p_{\mathrm{sat}}$, which, in turn, increases the driving force for bubble growth when the pressure inside the vessel is released. At the end of the foaming process, the foamed pellets are made to solidify and the cell number density is measured, being equal to 950/${\mathrm{cm}}^{3}$, 1050/${\mathrm{cm}}^{3}$, and 1300/${\mathrm{cm}}^{3}$ at $p_{\mathrm{sat}}$ = 4, 5, and 6 MPa, respectively. From the cell number density, the evolution of the average bubble radius ${\bar{r}}_{b}$ during the foaming process can be evaluated, which is shown in the inset in Figure 3b. The computation of ${\bar{r}}_{b}$ is based on two strong simplifying assumptions, namely, that the number of bubbles in the pellet is always equal to the final value (thus, neither coalescence nor break-up occur), and that the bubbles are spherical. Finally, examples of SEM images of the experimental foam morphology at increasing $p_{\mathrm{sat}}$ are reported in Figure 3c from left to right.

## 3. Direct Numerical Simulations

### 3.1. Mathematical Model

**Figure 4:**
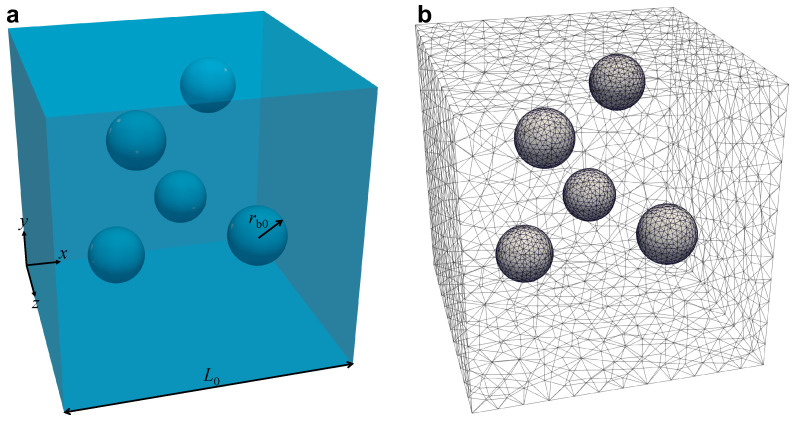
Scheme of the computational domain for direct numerical simulations (**a**) and of its discretization with a tetrahedral unstructured mesh (**b**).

In Figure 4, the computational domain considered in the direct numerical simulations (DNS) is displayed, namely, a cube, with initial side $L_{0}$, made of viscoelastic liquid. A Cartesian system of coordinates is set with the origin coinciding with a vertex of the cube. Five initially spherical gas bubbles with (equal) radius $r_{b0}$ are randomly distributed inside the cube. It is worth mentioning that the bubbles are ‘holes’ in the computational domain, namely, no balance equations are solved in the gas phase, as further detailed below.

Assuming the liquid phase to be isothermal, incompressible, and inertialess, its dynamics is governed by the mass and momentum balance equations in the Stokes formulation, reading

(1) ∇·u=0,

(2) ∇·T=0,

where u is the velocity vector and $T=-pI+2\eta_{s}D+\sum_{i=1}^{m}\tau_{i}$ is the stress tensor, with *p* the pressure, I the identity tensor, $\eta_{s}$ the Newtonian contribution to the liquid viscosity, $D=(\nabla u+\nabla u^{T})/2$ the rate-of-strain tensor, and $\sum_{i=1}^{m}\tau_{i}$ the viscoelastic extra-stress tensor, for which we consider a multi-mode constitutive equation with *m* modes. In particular, for each mode we use the Giesekus equation, reading

(3) $$\lambda_{i}{\overset{▽}{\tau }}_{i}+\tau_{i}+\frac{\lambda_{i}\alpha_{i}}{\eta_{p,i}}\tau_{i}^{2}=2\eta_{p,i}D,$$

with $\lambda_{i}$ the relaxation time, $\alpha_{i}$ the mobility parameter, and $\eta_{p,i}$ the polymer viscosity related to the *i*-th mode, respectively. The number of modes and the values of the rheological parameters considered in the DNS are obtained by fitting the experimentally measured values of the linear viscoelastic moduli $G^{\prime }$ and $G^{\prime \prime }$ and of the shear viscosity η of PCL at 35 °C shown in Figure 1. In particular, the regression yields m=3, whereas the values of $\eta_{s}$, $\lambda_{i}$, $\alpha_{i}$, and $\eta_{p,i}$, for *i* from 1 to 3, are reported in Table 1. As it is apparent by comparing the experimental points and the regression curves in Figure 1, a three-mode Giesekus constitutive equation allows to give an excellent description of the rheological behavior of the polymeric liquid employed in the experiments.

Strictly speaking, the bubbles grow due to the diffusion of the dissolved gas in the liquid phase, yet, for the sake of simplicity, in our description we neglect this, thus we do not include in the model the partial mass balance equation on ${\mathrm{CO}}_{2}$. Instead, we assume that there is a time-dependent pressure difference between the bubbles and the liquid far from them that makes them grow. This assumption enters the problem through the boundary condition on the interfaces between the bubbles and the surrounding liquid, which is

(4) $$T\;\cdot \;n=\gamma n\nabla \;\cdot \;n-p_{g}\left(t\right)n,$$

with n the unit vector normal to such interfaces and directed toward the liquid phase, γ=0.0193 Nm^−1^ the surface tension [6], and $p_{g}\left(t\right)$ the time-dependent gas pressure. In this regard, it is worth mentioning that $p_{g}\left(t\right)$ is imposed in the DNS by taking it from the extension of the single bubble growth model to the case of a multi-mode viscoelastic suspending liquid illustrated in Section 3.2. The fact that we impose on each bubble-liquid interface the same $p_{g}\left(t\right)$, taken from the single bubble growth model, implicitly contains the assumption that the bubbles do no interact. Such an assumption is altogether reasonable at the beginning of the bubble growth process, then, as the process goes on, it becomes patently excessive. However, even if the imposed $p_{g}\left(t\right)$ is derived by considering a simplified situation, hydrodynamic interactions among the bubbles mediated by the liquid are fully taken into account in the simulations, so our approach might represent a good compromise between the accuracy of the mathematical description of the phenomenon and the feasibility of the DNS.

**Figure 5:**
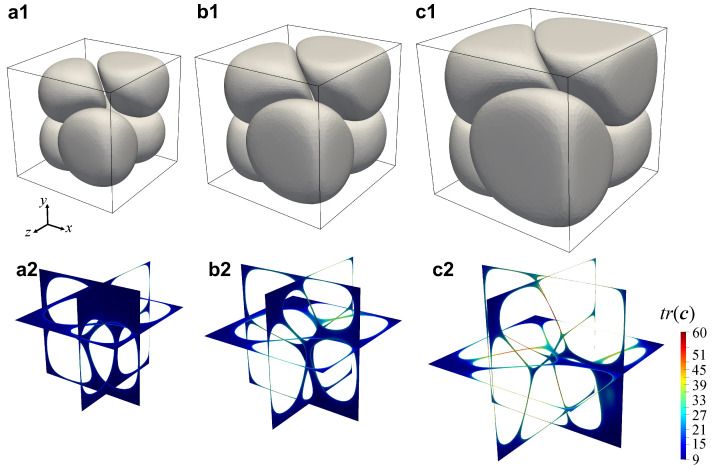
Numerically computed morphology of the foam (top row) and map of the trace of the conformation tensor tr(c) on three orthogonal cut planes (bottom row) at t=1.25s and $p_{\mathrm{sat}}$ = 4 (**a**), 5 (**b**), 6 MPa (**c**).

A periodicity condition is imposed on the external boundaries of the domain, meaning that the computational domain is a portion of space in the bulk of the foaming pellet, thus it ‘sees’ around it other portions where the process happens analogously. In this regard, it is worth remarking that the number of bubbles appearing in the computational domain is sufficient to ensure ‘bulky’ conditions. Indeed, tests have been made with a double number of bubbles, of course keeping the same volume concentration, yielding the same results, in terms of relative inflation of the system and stress fields, as those presented in this work. In addition, a sensitivity analysis has been done on the initial random distribution of the bubbles in the computational domain, showing that no significant modifications of the numerical results in Figure 3 and Figure 5 occur when the initial bubble configuration changes.

### 3.2. Single Bubble Growth

The dynamics of a spherical bubble growing in a viscoelastic liquid due to the presence of a supersatured gas dissolved into it is governed by the momentum balance equation for the polymeric matrix and by the diffusion equation for the gas. The model presented below follows the derivation proposed by Everitt et al. [18] and Tammaro et al. [6]. A single spherical bubble with initial volume $V_{0}=4/3\pi R_{0}^{3}$ ($R_{0}$ being, of course, the initial radius) is surrounded by a spherical shell of an incompressible viscoelastic liquid containing a dissolved gas at initial pressure $p_{g0}$. The pressure of the external ambient is $p_{a}$ and the pressure difference $p_{g}\left(t\right)-p_{a}$ drives the bubble growth. A spherical system of coordinates with origin at the center of the bubble is considered. Due to the liquid volume conservation, it is convenient to transform the radial coordinate *r* into a Lagrangian volume coordinate $x=r^{3}-R^{3}$ [18], with *R* the time-varying bubble radius. The outer edge of the liquid domain is at x=X. Hence, the volume of the shell is given by 4/3πX. At the outer shell boundary, the liquid normal stresses are balanced by the ambient pressure $p_{a}$. At the gas-liquid interface, the normal stresses are equal to the bubble pressure $p_{g}$ plus the contribution of the surface tension. We assume: (i) isothermal conditions, (ii) negligible inertia, (iii) validity of the Henry’s law at the gas-liquid interface. The rheological behavior of the viscoelastic liquid is modeled by the multi-mode Giesekus constitutive equation. Under these assumptions, the equations governing the bubble dynamics are

(5) $$\frac{4}{3}\eta_{s}\dot{u}\left(\frac{1}{u}-\frac{1}{u+X}\right)=p_{g}-p_{a}+\frac{2}{3}\sum_{i=1}^{m}\int_{0}^{X}\frac{T_{rr,i}-T_{\theta \theta ,i}}{x+u}dx-\frac{2\gamma }{u^{1/3}},$$

(6) $$\lambda_{i}\frac{\partial \tau_{rr,i}}{\partial t}+\left(\frac{4\dot{u}\lambda_{i}}{3(x+u)}+1\right)\tau_{rr,i}+\frac{\alpha_{i}\lambda_{i}}{\eta_{p,i}}\tau_{rr,i}^{2}=-\frac{4\dot{u}}{3(x+u)}\eta_{p,i},$$

(7) $$\lambda_{i}\frac{\partial \tau_{\theta \theta ,i}}{\partial t}-\left(\frac{2\dot{u}\lambda_{i}}{3(x+u)}-1\right)\tau_{\theta \theta ,i}+\frac{\alpha_{i}\lambda_{i}}{\eta_{p,i}}\tau_{\theta \theta ,i}^{2}=\frac{2\dot{u}}{3(x+u)}\eta_{p,i},$$

(8) $$\frac{\partial \phi }{\partial t}=9D{\left(x+u\right)}^{4/3}\frac{\partial^{2}\phi }{\partial x^{2}},$$

(9) $$p_{g}u=p_{g0}u_{0}+RT\phi \left(0,t\right).$$

Equation (5) expresses the momentum balance in the liquid, where $u\left(t\right)=R^{3}\left(t\right)$ is proportional to the bubble volume, $\eta_{s}$ is the Newtonian contribution to the liquid viscosity, *m* is the number of modes, and $T_{rr,i}$ and $T_{\theta \theta ,i}$ are the rr- and θθ-components of the *i*-th mode of the stress tensor, respectively. The evolution of these two stress components for a liquid obeying the Giesekus constitutive equation is expressed in Equations (6) and (7), where $\lambda_{i}$, $\eta_{p,i}$, and $\alpha_{i}$ are the relaxation time, the polymer viscosity, and the ‘mobility’ parameter for the *i*-th mode, respectively. Equation (8) is the diffusion equation, where, for numerical reasons [18], a concentration potential ϕ(x,t) such that $\partial \phi /\partial x=c-c_{0}$ is introduced, with *c* the gas concentration and $c_{0}$ its initial value. This equation is solved with boundary conditions $\partial \phi /\partial x=(p_{g}-p_{g0})H$ at the bubble surface, *H* being the Henry’s constant, and $\partial^{2}\phi /\partial x^{2}=0$ at the outer edge. Finally, Equation (9) stems from the conservation of the mass in the bubble, with *R* and *T* the universal gas constant and the temperature. These equations are supplied with the initial conditions $u\left(0\right)=u_{0}$, $T_{rr,i}=T_{\theta \theta ,i}=0$, ϕ(0)=0.

The number of modes, m=3, and the values of the constitutive parameters of the liquid are obtained by regression of the PCL rheological data, yielding the values reported in Table 1. The temperature is set to T=35 °C. The diffusivity, surface tension, and Henry’s constant are $D=3.5\times 10^{-10}$ m^2^s^−1^, γ=0.0193Nm^−1^, and $H=5.2\times 10^{-5}\;\mathrm{mol}$N^−1^m^−1^, respectively [6]. The initial gas pressure $p_{g0}$ is set according to the experimental values of $p_{\mathrm{sat}}$, the initial bubble volume $u_{0}$ is chosen such that $R_{0}=100\;\mu m$ (correspondingly, the same value is given to $r_{b0}$ in the DNS), and the fluid volume *X* surrounding the bubble is selected such that the pressure evolution predicted by the single bubble growth model at $p_{g0}=5\mathrm{MPa}$ is close to the experimental trend shown in Figure 3a (*X* is kept the same also when considering $p_{g0}$ = 4 and 6 MPa). From this value, the value of $L_{0}$ employed in the DNS is obtained by taking a volume of the computational domain five times the volume considered in the single bubble growth model, yielding $L_{0}=9.6\times 10^{-4}\;m$.

Equations (5)–(9) are solved through the method of lines. The evolution of the quantity $(p_{g}-p_{a})4/3\pi u$ is computed and used to derive the time-depending boundary condition at the bubble-liquid interfaces in the DNS.

### 3.3. Numerical Technique

The equations constituting the mathematical model reported above are solved through the arbitrary Lagrangian Eulerian (ALE) finite element method (FEM). The outcomes of our numerical simulations are the full velocity and stress fields dynamics in the whole liquid domain, as well as the morphological evolution of the gas bubbles. As said above, the pressure making the bubbles grow is given as an input at the gas-liquid interfaces, so the velocity and stress fields in the gas phase are not solved. As reported at the beginning of Section 3.1, the computational domain is a cube of initial side $L_{0}$ with five initially spherical ‘holes’ of radius $r_{b0}$, i.e., the gas bubbles. The liquid phase is discretized by means of a mesh made of quadratic tetrahedra, as shown in Figure 4b. We use quadratic ($P_{2}$) interpolation for the velocity u and linear ($P_{1}$) interpolation for the pressure *p* (Taylor-Hood elements).

During the evolution of the process, the deformable boundaries of the gas bubbles need to be tracked: to do this, a FEM with second-order time discretization is defined on such surfaces, where the normal component of the mesh velocity equals the normal component of the physical velocity, whereas the tangential velocity of the mesh nodes is such that the distribution of the elements on the interfaces is optimized. Compared to a Lagrangian one, this approach greatly reduces the distortion of the mesh on the surfaces of the bubbles. Details can be found in Villone et al. [19].

Bubble inflation makes them approach each other and squeeze the liquid in between them, making the mesh tetrahedral elements progressively deform. Every time the mesh quality, in terms of the aspect of the ‘worst’ element in the domain, goes below a given threshold, a remeshing is performed and the solution is projected from the old mesh to the new one [20,21]. Moreover, since great deformations arise when the bubbles are close to each other, mesh refinements are performed when necessary, thus ensuring the presence of a minimum number of volume elements in the liquid films between the bubbles. It is worth remarking that, like all the sharp-interface methods, the technique used in this work does not allow to deal with bubble coalescence. On the other hand, it allows to give a very accurate description of the stress fields in the thin layers in between the bubbles, which is a relevant quantity for the prediction of the foam morphology, as discussed in Section 3.4.

### 3.4. Numerical Results

The solid lines in Figure 3b report the temporal history of the computational domain volume *V*, normalized by the initial value $V_{0}$, during the foaming process at $T_{\mathrm{foam}}=35$ °C and $p_{\mathrm{sat}}=4,5,6\;\mathrm{MPa}$ (see legend). It is apparent that, for each $p_{\mathrm{sat}}$-value, a fair quantitative agreement exists between the experimental and the numerical data. Hence, the same comments can be made, i.e., that, the larger $p_{\mathrm{sat}}$, the faster *V* increases. It is worth remarking that the computational curves stop earlier than the experimental data because the adopted numerical technique is not capable to deal with topological changes, namely, when the thickness of the liquid film separating the bubbles becomes too little and the bubbles coalesce.

In the first row of Figure 5, we report the numerically computed morphology of the foam at t=1.25s and $p_{\mathrm{sat}}$ = 4 (a1), 5 (b1), and 6 MPa (c1), showing that, at a given time, the larger $p_{\mathrm{sat}}$ the larger the computational volume due to a larger bubble growth. It can be observed that, at t=1.25s, the shapes of the bubbles are at this point very far from the initial spherical ones due to the hydrodynamic interactions among them. In particular, the facing portions of the surfaces of approaching bubbles flatten and the liquid film in between them gets squeezed, possibly undergoing a rupture if its thickness goes below a critical dimension. If this happens, the broken liquid wall might retract depending on the amount of elastic energy that it has stored during bubble growth. Whether retraction occurs or not will, in turn, determine if the foam will have an open-cell or a closed-cell morphology [6]. Given the good agreement between the experimental and the numerical results, the latter can be indeed used to investigate quantities hardly attainable otherwise, as the aforementioned amount of elastic energy is stored inside the liquid, which can be measured by looking at the trace of the conformation tensor $c=\sum_{i=1}^{m}\left[\left(\lambda_{i}/\eta_{p,i}\right)\tau_{i}+I\right]$ [22]. In the second row of Figure 5, we display the map of the trace of the conformation tensor tr(c) computed on three orthogonal cut planes at t=1.25 s and $p_{\mathrm{sat}}$ = 4 (a2), 5 (b2), and 6 MPa (c2), showing that, at such given time, the larger $p_{\mathrm{sat}}$ the thinner the liquid films in between the growing bubbles and, correspondingly, the larger the amount of elastic energy they contain. In particular, it can be observed that, at $p_{\mathrm{sat}}=4\;\mathrm{MPa}$, tr(c) is almost everywhere close to 3, namely, the bubble growth is slow compared to the liquid relaxation time, thus the polymeric matrix is able to relax the elastic stress while bubbles inflate. As a consequence, little or no retraction is expected if the liquid wall between two bubbles breaks, yielding a closed-cell foam or a foam with interconnected cellular structure. On the contrary, at $p_{\mathrm{sat}}=6\;\mathrm{MPa}$, tr(c) attains much larger values, of about 60, in the thin films between the bubbles, thus the bubble growth is fast compared to the liquid relaxation time and the polymeric matrix is no longer able to relax the elastic stress while bubbles inflate. Hence, a complete retraction is expected if the liquid wall between two bubbles breaks, yielding an open-cell foam. At $p_{\mathrm{sat}}=5\mathrm{MPa}$, intermediate values of tr(c) can be observed in the films among the bubbles, suggesting that an intermediate scenario between the two described above could occur as a consequence of bubble wall rupture.

## 4. Conclusions

In this paper, we study experimentally the influence that ${\mathrm{CO}}_{2}$ saturation pressure has on the growth of foamed PCL pellets obtained by gas foaming technology, showing that, given all the other process and constitutive parameters, the volume of the foamed pellet increases faster and to a larger final value by increasing the saturation pressure. The experimental data are compared with the outcomes of three-dimensional finite-element direct numerical simulations of bubble inflation in a three-mode Giesekus viscoelastic liquid describing the rheology of the experimentally employed PCL. These allow us to give a very accurate description of the stress fields in the thin liquid films in between gas bubbles as they approach each other, showing that the larger the saturation pressure, the faster the bubble growth, and thus the larger the amount of elastic energy accumulated in the liquid, which is a driving force for bubble wall retraction in the case of rupture. Hence, numerical simulations provide us with a tool to predict foam morphology. Since a suitable bubble growth law is required as a simulation input, we extend the analytic model available in the literature [6,12] for the growth of a single bubble in a liquid to the case of a multi-mode viscoelastic liquid.

## Figures and Tables

**Table 1:** Values of the rheological parameters employed in the DNS, obtained by fitting the experimental measurements reported in Figure 1 with a three-mode Giesekus model.

*η*_s_ [Pa s^−1^]	5691.58 Pa s^−1^		
*i*	1	2	3
$\lambda_{i}\left[s\right]$	4.13	0.52	0.083
$\alpha_{i}$	0.82	0.53	0.48
$\eta_{p,i}$[Pa s^−1^]	32,258.0	41,419.0	27,964.0
